# Peri-Traumatic Distress and Its Relationship to Resilience and Coping Among Medical Students in Malaysia During COVID-19 Lockdown

**DOI:** 10.3389/fpsyt.2021.789554

**Published:** 2021-12-06

**Authors:** Salina Mohamed, Zaliha Ismail, Norley Shuib, Nur Faizah Ali

**Affiliations:** ^1^Department of Psychiatry, Faculty of Medicine, Universiti Teknologi MARA, Sungai Buloh, Malaysia; ^2^Department of Public Health Medicine, Faculty of Medicine, Universiti Teknologi MARA, Shah Alam, Malaysia

**Keywords:** medical students, undergraduate, peritraumatic, distress, coping, resilience, COVID-19, lockdown

## Abstract

**Background:** Medical students are not spared from the challenges related to the Covid-19 lockdown. The fear and uncertainties may lead to traumatic symptoms and test their resilience and sense of coping. Thus, this study aims to determine the prevalence of peri-traumatic distress symptoms and its association with the level of resilience and the coping strategies used during the lockdown among medical students.

**Materials and Methods:** This was a cross-sectional online questionnaire survey involving medical students from a public university in Malaysia. It was conducted during the COVID-19 lockdown or Movement Control Order (MCO) where the students were asked to fill in the COVID-19 Peri-traumatic Distress Index (CPDI), Brief COPE Inventory, and Connor Davidson Resilience Scale (CDRS-25).

**Results:** A total of 282 clinical and 172 pre-clinical medical students were involved. Peri-traumatic distress symptoms were reported by 27% out of the total students. This study found that those who were having peritraumatic distress symptoms were from the pre-clinical years, had poor internet access, as well as lower resilience levels, and used more dysfunctional coping strategies. Pre-clinical medical students with difficult internet access were eight times more likely to have peritraumatic distress symptoms.

**Conclusions:** Given the high level of peri-traumatic distress symptoms reported by medical students during the lockdown, it is vital to identify the vulnerable students, assess their needs and risks to mental health problems during this challenging time as the pandemic is still ongoing with countries going in and out of lockdown depending on the cases at the time. The university administration for each University in Malaysia will need to have a clear academic guideline and policy as well as providing improved infrastructure to minimize the distress faced by medical students.

## Introduction

The COVID-19 pandemic posed unique challenges to everyone in the world. It affects the country and the community in various aspects such as its economy, putting increasing strains on resources, particularly the healthcare system, and renders other areas such as the educational system to a halt. COVID-19 is a global pandemic that challenges an individual's mental health, coping abilities, and resilience. During a pandemic, the individual's resilience might be tested, and it may cause various psychological distress reactions, risky behaviors, and even psychiatric disorders (1). The COVID-19 lockdown has been shown to have an adverse effect on people in the community. A study comparing the mental wellbeing of people in lockdown in Asia, Africa, and European countries showed that it had a negative effect on both mental wellbeing and mood, particularly depressive symptoms during the lockdown as compared to before the lockdown (2). Banerjee et al. (3) in a systematic review on the psychological impact of lockdown in the South-Asian population, reported an increase in depression, anxiety, somatic concerns, alcohol-related disorders, and insomnia.

In Malaysia, the COVID-19 lockdown or Movement Control Order (MCO) started from the middle of March 2020 until the middle of May 2020. All education centers were closed immediately and for students, regardless of whether they are in primary, secondary, or tertiary education level, being at home due to the movement control order created many challenges to the students and families as well. The university students were instructed to return home immediately upon announcement of the MCO by the Malaysian Government and online classes were commenced. The abrupt change learning method coupled with the COVID-19 related fear and lockdown created many challenges for undergraduate students. COVID-19 specific worries, isolation in social networks, lack of interaction and emotional support, and physical isolation were associated with poorer mental health in students (4). Students reported academic and everyday difficulties with high levels of mental health distress where levels of depression and anxiety were higher during the lockdown and these were associated with the inability to focus on studies and loss of employment (4–6).

Medical students were not spared from such challenges. Peri-traumatic distress is emotional and physiological distress experienced during and/or immediately after a traumatic event and is associated with the development and severity of posttraumatic stress disorder (PTSD) and related psychological difficulties. Medical students' perception and definition of risk and danger to themselves and/or to their families may have changed during this pandemic. These coupled with the fear and uncertainties may lead to traumatic symptoms and cause challenges to their usual coping abilities and resilience. Their concerns of continuing medical studies, the lack of routine and schedules, loss of social connections, and being in isolation put them at risk of developing further psychological distress such as depression and anxiety (7–9). The pandemic may give rise to peri-traumatic distress to medical students and lockdown as a traumatic experience has not been explored in medical students. As these peri-traumatic distress symptoms may be a precursor to depression and anxiety, the University's mental health services need to be aware of these issues and plan for prevention, active screening, and intervention to the medical students who are currently struggling.

Resilience and having good coping strategies are important in mitigating the stressful impact and peri-traumatic distress of lockdown. Globally, studies have shown that university students have employed several coping strategies during the lockdown which included seeking social support, avoidance through technology and playing video games, cognitive strategies, and religion (10–14). Resilience is seen as the ability to have psychological flexibility and the emotional capability to deal and adapt to difficult or traumatic life challenges whilst maintaining a meaningful quality of life. The ability to recover from an adverse event is also determined by a person's resilience (15). A study done on young adults between 18–25 years old found that the relationship between stress, coping and resilience during the pandemic lockdown was complicated (16). A good level of resilience seemed to protect against developing some mental disorders but not with others. Having a high resilience level among university students was associated with lower rates of depression and anxiety during the pandemic but not protective against post-traumatic distress disorder (17). However, another study done on the role of psychological flexibility and inflexibility during the pandemic lockdown fond that having psychological flexibility protects against peri-traumatic distress, depression and anxiety (18). To the authors knowledge, there has been no studies that investigated the association between peri-traumatic distress with coping and resilience among medical students or other faculties done to date.

Therefore, this study aimed to ascertain the prevalence of peri-traumatic distress symptoms among medical students and its association with the level of resilience of medical students and the coping strategies used during the lockdown in Malaysia.

## Materials and Methods

This study was a cross-sectional online questionnaire survey involving medical students from a public university in Malaysia. It was conducted during the COVID-19 lockdown or Movement Control Order (MCO) in Malaysia which started from the middle of March 2020 until the middle of May 2020.

### Participants and Study Procedure

The questionnaires were sent to all 1125 medical students from Year 1 and Year 2 (pre-clinical) and Year 3 to 5 (clinical) via Whatsapp software. The design of the survey allows us to take consent before the respondent can fill in the questionnaires. The questionnaires were comprised of sociodemographic and MCO-related details, COVID-19 Peri-traumatic Distress Index (CPDI), Brief COPE Inventory, and Connor Davidson Resilience Scale (CDRS-25).

Those who scored positive as having distress will be contacted for further assessment once consented. A minimum of 350 respondents was needed and at the end of the study, 454 medical students responded. The students received a cover letter prior to the answering the questionnaires reassuring them about anonymity, confidentiality, and that published results were solely for scientific purpose. Ethical approval was obtained from the University's Research Ethics Committee REC/07/2020 (MR/167).

### Measures

#### COVID-19 Peri-Traumatic Distress Index (CPDI)

It was adapted from The Peri-traumatic Distress Scale (PDI) that was used to assess emotional, cognitive, and physical reactions occurring during a critical incident and immediately after. The COVID-19 Peri-traumatic Distress Index (CPDI) (19) assesses the frequency of anxiety, depression, specific phobias, cognitive change, avoidance, and compulsive behavior, physical symptoms, and loss of social functioning in the past week, ranging from 0 to 100. A score between 28 and 51 indicates mild to moderate distress. A score of 52 and above indicates severe distress. The Cronbach's alpha of CPDI was 0.95 (*p* < 0.001). The total raw score should be increased by 4 to make it 100 (Display score). The displayed score will be further categorized into Normal: 0–28, Mild: 29–52, Severe: 53–100 psychological distress.

#### Brief COPE Inventory

It was created by Carver (20) and was an abbreviated version of the full COPE Inventory. This questionnaire aims to assess the type of coping behavior using 14 coping strategies. Two items in the list represent these coping strategies: self-distraction, active coping, denial, substance abuse, emotional support, instrumental support, behavioral disengagement, venting, positive reframing, planning, humor, acceptance of religion, and self-blame. Each item was accompanied by a 4 point Likert scale with a score of 0 as 'I haven't been doing this at all', a score of 1 as 'I've been doing this a little bit', a score of 2 as 'I've been doing this a medium amount' and a score of 3 as 'I've been doing this a lot'. Items that scored a value of ≤ 1 were converted to a value of 0 to signify that the particular coping strategy was not performed at all, whereas items that scored a value of ≥2 were converted to a value of 1 to signify otherwise. The total score for a particular coping method was accumulated together and interpreted as follows: a score of 0 signified that the students did not perform the coping method at all, a score of 1 signified that the student had performed at least one out of two of the particular coping method and a score of 2 signified that the students had performed both of the coping methods. This questionnaire has been commonly used to determine the common coping behaviors in several studies involving medical students (21, 22).

#### Connor Davidson Resilience Scale (CDRS-25)

It was originally developed as a self-report measure of resilience and (23). CDRS−25 has been validated for the Malaysian population and widely used resilience scale where the 25-item scale has the total possible scores range from 0–100. It measures several components of resilience: the ability to adapt to change, the ability to deal with what comes along, the ability to cope with stress, the ability to stay focused and think clearly, the ability to not get discouraged in the face of failure, the ability to handle unpleasant feelings such as anger, pain or sadness. The higher the score, the higher the resilience level.

### Statistical Analysis

All the responses will be collected in a spreadsheet and transferred to IBM SPSS version 25.

CPDI: Mean (SD) scores for total and each category will be calculated.

Brief COPE: The association between the category of sociodemographic factors and severity of psychological stress will be analyzed by chi-square test. Multinomial logistic regression analysis will be performed to determine the sociodemographic factors associated with psychological distress.

CDRS-25: The total possible scores range from 0–100. The higher the score, the higher the resilience level. The cut-off point is determined from the mean.

## Results

A total of 454 medical students responded to the questionnaires of which 62.1 and 37.9% were pre-clinical and clinical, respectively. Table 1 shows the medical students' profiles and COVID-19 related data.

**Table 1:** Undergraduate medical student profiles and Covid-19 related data.

	**N**	**(%)**
**Students**		
Pre-clinical	282	62.1
Clinical	172	37.9
Gender		
Female	363	80.0
Male	91	20.0
Internet accessibility		
Yes	408	89.9
Difficult	46	10.1
Staying with during MCO		
Family	448	98.9
Not with Family	6	1.1
Time spent on COVID-19 related news/day		
<30 min	296	65.2
1–2 h	135	29.7
3–4 h	16	3.5
>5 h	7	1.5
Frequency of contact with people other than family		
Once/week	217	47.8
Twice/week	44	9.7
Three times/week	61	13.4
Everyday	120	26.4
People contacted during lockdown		
Other family members	38	8.4
Coursemate	26	5.7
Other friends	16	3.5
Lecturers	3	0.7
Knew someone who is Person Under Investigation (PUI)		
No	400	88.1
Friend	21	4.6
Self	10	2.2
Family	14	3.1
Knew someone who is COVID-19 positive		
No	412	90.7
Friend	27	5.9
Family	7	1.5
Higher resilience ≥64	227	50.0
Lower resilience <64	219	48.2

Among the 454 who responded, 439 students completed the CPDI scale. The prevalence of peri-traumatic distress among the medical undergraduates was 27% (*N* = 439). Table 2 showed that a significantly higher proportion of distress was found among the pre-clinical students, and in terms of sociodemographic and COVID-19 related variables, female students were more distressed as compared to their male counterparts. All other variables were not statistically significant.

**Table 2:** Factors associated with peritraumatic distress among medical students.

**Variables**	**CPDI** **N(%)/Mean (SD)**		**X^2^ value/t stat**	***P*** **value**
	**Distress**	**Normal**		
Student				
Pre-clinical	89(32.2%)	187(67.8%)	11.903	**0.001**
Clinical	28(17.2%)	135(82.8%)		
Gender				
Female	86(24.6%)	264(75.4%)	3.821	0.050
Male	31(34.8%)	58(65.2%)		
Internet access				
Yes	99(25.4)	294(74.8)	4.093	0.043
Difficult	18(39.4)	28(60.9)		
Resilience level				
Higher resilience ≥64	30(13.7)	189(86.3)	36.82	**<0.001**
Lower resilience <64	84(39.4)	129(60.6)		
CDRS25 total score	56.8(14.01)	66.9(13.38)	6.794	<0.001^*^

Pearson chi-square ^*^independent t-test. Items in bold are statistically significant, p < 0.05.

Out of seven variables included in simple logistic regression, there were four variables significantly associated with peri-traumatic distress (Table 3). All the five significant variables (pre-clinical, female, difficult internet access, low resilience, dysfunctional coping strategies) from simple logistic regression were then included in multiple logistic regression. However, in multiple logistic regression, female gender was not significantly associated (Table 4). Those in pre-clinical years were 2.14 times more likely to have peri-traumatic distress as compared to those in clinical years. Those who have difficulty with internet access were 2.69 times more likely to be distressed as compared to those who do not have difficulty with internet access. Medical students with lower resilience levels were 3.32 times more likely to have peri-traumatic distress symptoms as compared to those with higher resilience. This study also found that students who used dysfunctional coping strategies were 1.29 times more likely to have peritraumatic distress symptoms. Pre-clinical medical students with difficult internet access were 7.93 times more likely to have peritraumatic distress during the lockdown.

**Table 3:** Factors associated with peri-traumatic distress using Simple logistic Regression.

	**B**	**SE**.	**Wald**	**OR (95%CI)**	***P*** **-value**
Female	0.680	0.340	3.999	1.97(1.014,3.840)	**0.046**
Pre-clinical	0.826	0.290	8.121	2.29(1.294,4.033)	**0.004**
Difficult Internet access	0.974	0.426	5.222	2.65(1.149,6.103)	**0.022**
CDRS Low resilience	1.183	0.318	13.790	3.26(1.748,6.092)	**<0.001**
Dysfunctional coping strategies	0.287	0.042	46.581	1.33(1.227,1.448)	**<0.001**
Problem focused coping strategies	−0.089	0.055	2.604	0.92(0.822,1.019)	0.107
Emotion focused coping strategies	0.039	0.040	0.986	1.04(0.962,1.124)	0.321

**Table 4:** Factors associated with peri-traumatic distress using Multiple logistic Regression.

	**B**	**SE**.	**Wald**	**AOR (95%CI)**	***P*** **-value**
Pre-clinical	0.760	0.276	7.601	2.14 (1.246, 3.673)	**0.006**
Difficult internet access	0.991	0.403	6.051	2.69(1.223,5.928)	**0.014**
Low resilience	1.199	0.282	18.081	3.32(1.909,5.767)	**<0.001**
Dysfunctional coping strategies	0.262	0.034	60.698	1.29 (1.216,1.387)	**<0.001**
Pre-clinical with Difficult internet access	2.07	0.538	14.791	7.93(2.760, 22.758)	**<0.001**

*^a^Statistical test: Multiple logistic Regression. AOR (adjusted odds ratio) is statistically significant at α = 0.05; the Model is fit, multicollinearity, the interaction of pre-clinical years, and difficult internet access is significant*.

## Discussion

The purpose of this study was to assess the prevalence of peri-traumatic distress symptoms among medical students and its association to the level of resilience and coping strategies used during the COVID-19 lockdown period. The COVID-19 Peri-traumatic Distress Index (CPDI) measures the frequency of anxiety, depression, specific phobias, cognitive change, avoidance, and compulsive behavior, physical symptoms, and loss of social functioning in the past week.

From this study, the prevalence of peri-traumatic distress symptoms among medical students during Covid-19 lockdown as measured using CPDI was found to be 26.7%. This is higher than the finding reported by (24) where the study found 13.2% of their participants had PTSD-positive symptoms as measured by Posttraumatic Stress Disorder Checklist-5, adapted to measure pre/peri/posttraumatic reactions in relation to COVID-19. In studies looking for psychological distress among university students, the finding is lower compared to the prevalence of anxiety in a study conducted among 16 university students which found that 56 % of students suffered from anxiety (25). However, the prevalence is higher than the prevalence in a study conducted by Sundarasen et al. (26) which found only 8% of students suffered from anxiety in the current COVID-19 situation. A cross-sectional web-based study conducted among university students across Bangladesh reported that 15% had moderately severe depression, whereas 18.1% were suffering from severe anxiety during the COVID-19 pandemic (6). However, this study used different tools to assess psychological distress, the Patient Health Questionnaire (PHQ-9) and Generalized Anxiety Disorder (GAD-7) questionnaires for the assessment of depression and anxiety, respectively. In a different study, Swiss undergraduate students under lockdown experienced worsening of stress, anxiety, loneliness, and depressive symptoms compared to before the period (4). The unprecedented nature of the COVID-19 Movement Control Order (MCO) in Malaysia has disrupted the daily routines and affected the psychological wellbeing of medical students in various aspects. Standard Operating Procedures (SOPs) or restrictions imposed during the MCO have negatively affected the freedom and autonomy in making simple daily decisions like leaving the house or getting together with friends. Young adults were struggling to cope with feelings of loneliness and isolation as a result of physical distancing from family and friends due to the lockdown. Sense of being unproductive, helplessness, and a loss of a sense of purpose were also experienced in this group. They also reported feelings of anxiety, sadness, and agitation (27).

A significantly higher proportion of pre-clinical students (32.2%) experienced peri-traumatic distress symptoms compared to their clinical year counterparts (17.2%) during the MCO period. This is comparable to the findings from a previous study conducted in Lebanon (62%) which found a high level of stress among pre-clinical students (28). Academic factors that have been identified to be related to stress among medical students are academic demands, medical syllabus, exams, excessive workload, and the learning environment. Whereas individual factors that contributed to stress among these groups of students are the inability to cope, feelings of helplessness, increased emotional pressure and mental tension, and personal life events (29). During the COVID-19 pandemic, these students had to adapt to new changes in curricular delivery which mainly focused on online distance learning (ODL). Furthermore, the Year 1 students did not have any face-to-face contact with their educators and friends upon enrolment to the medical program to familiarize themselves with the new learning and teaching environment. This posed some challenges to establish an adequate support system within the program itself.

In this study, there is an association between difficult internet access and peritraumatic distress among medical students, and this association was almost eight times more likely in those in the pre-clinical years. This is consistent with a study done in Bangladesh in which students were very much affected psychologically due to poor and limited internet access (30). Since COVID-19 started last year, like any other part of the world, the University took an active initiative to adapt its teaching and learning online. Various factors posed challenges to online learning, such as the quality of virtual content, updates on technology, technical assistance, and interaction with other course mates (31). The students depend on the availability and efficiency of the internet at home, and unfortunately, not all areas in Malaysia have good internet coverage. In addition that, the students possibly could not afford a better internet connection due to the monthly charges. This had caused the students to face difficulties in participating in online classes and discussions. Assessments and exams were also conducted online. Due to this, they might be in distress thinking if they had optimum training or if anything would go faulty during their exams if the internet connection is difficult. Due to restricted movement during the lockdown, most students resort to virtual social interaction through social media or online activities such as gaming. A study by Alkan and Meinck (32) showed the importance of information technology in social communication in daily life, which is more relevant in the current pandemic situation. Therefore, the limitation of the internet connection could hinder this process which can cause psychological distress in these students.

Dysfunctional coping skills were significantly associated with peri-traumatic distress in this study whereby the students who employed this type of coping skills were more likely to become distressed. This concurs with the findings from previous studies that having dysfunctional coping skills not only will cause distress but also causes inefficiency to tackle the source of the stress, unlike those who use emotion-focused or problem-focused coping skills (33, 34). In the current COVID-19 pandemic, avoidant coping behavior which is part of dysfunctional coping skills was positively associated with distress and detrimental to psychological wellbeing (35). It is likely that the medical students who were distressed did not have better coping skills in dealing with their daily circumstances and stress. Likely the medical students adopt the dysfunctional coping styles because of their lack of experience as most of them are still early in their training to be doctors and have not developed the maturity to process their emotions and behavior to cope better. The lack of proper training of healthy coping skills in the formal curriculum in Medicine too can serve as the contributing factor of them lacking the appropriate skills. To the authors' knowledge, there is also no specific course incorporated in the curriculum of local medical schools, or in fact, medical schools in other Asian countries, that promotes self-health and wellness, although these skills are vital to cultivate the behavior that ensures the resilience throughout their lifetime career. Such curriculum had been introduced in most medical schools in the United States (36). During this pandemic, psychological flexibility has been associated with better coping and resilience level. It has been found to lower the risk of peri-traumatic distress, depression and anxiety (18).

Medical students who are more resilient have lower odds of experiencing peri-traumatic distress symptoms. This finding is similar to previous studies conducted among university students which concluded that resilience is vital in experiencing less psychological distress as it is a stable trait and negatively correlates with distress (37, 38). Hence it is important for educators to help in building and strengthening resilience among medical students to face the challenges and uncertainties posed by the COVID-19 pandemic. Resilience can be built or enhanced by the four main principles of being in control (being in control and calm when faced with difficulties), being involved (dedicated to deal with difficulties), being resourceful (capacity to find alternatives to deal with difficulties), and being “growth”-centric (ability to keep growing and bounce back better when faced with difficulties) (39).

## Strengths and Limitations

To the knowledge of the authors, this is the first study being conducted assessing peri-traumatic distress among medical students in Malaysia during the COVID-19 lockdown. It was conducted in a timely manner. However, this study poses a few limitations. Firstly, this is a cross-sectional study. Therefore, only factors associated with peri-traumatic distress can be assessed instead of the causal linkage. Secondly, the universal (non-probability) sampling method that had been used can lead to bias that will affect the accuracy of the result. Thirdly, participants in this study were limited to the involvement of medical students in one public University in Malaysia. It did not represent the whole population of tertiary education in Malaysia. In this study, the evaluation was done using self-reported questionnaires. Some forms of biases that could happen were recall bias, respond-bias, honesty, and the medical students' understanding of the questionnaires.

Lastly, the questionnaire served as a screening tool for peri-traumatic distress among medical students. It was not diagnostic in nature. In the future study, diagnostic tools can be used so that mental illness among the medical students can be detected, and referral or intervention can be offered.

## Conclusions

In order to face the many challenges and the dynamic changes posed by this pandemic, medical students need to develop healthy coping skills and improve their resilience in order to bounce back stronger to continue on with their medical training. It is essential for the university administration to identify the vulnerable students, assess their needs and risks to mental health problems during this challenging time. One of the ways is for the University or faculty to provide good psychological support for their students, for example, by providing a hotline which the students can contact anytime they need to. Coping skills and resilience training, and psychological support groups for those in need can also be provided virtually so that students can utilize them wherever they are. Improving the infrastructure, such as better internet services on the campus, would be beneficial. The importance of constant and effective communication between the University and the students with clear guidelines and policies should not be undermined as it is empirical in alleviating the distressing effect of COVID-19.

## Data Availability Statement

The raw data supporting the conclusions of this article will be made available by the authors, without undue reservation.

## Ethics Statement

The studies involving human participants were reviewed and approved by UiTM Research Ethics Committee REC/07/2020 (MR/167). The patients/participants provided their written informed consent to participate in this study.

## Author Contributions

All authors listed have made a substantial, direct, and intellectual contribution to the work, and approved it for publication.

## Conflict of Interest

The authors declare that the research was conducted in the absence of any commercial or financial relationships that could be construed as a potential conflict of interest.

## Publisher's Note

All claims expressed in this article are solely those of the authors and do not necessarily represent those of their affiliated organizations, or those of the publisher, the editors and the reviewers. Any product that may be evaluated in this article, or claim that may be made by its manufacturer, is not guaranteed or endorsed by the publisher.
